# Supplementary material for: The Antihypertensive Effects and Safety of LCZ696 in Patients with Hypertension: A Systemic Review and Meta-Analysis of Randomized Controlled Trials
Source: J Clin Med. 2021 Jun 26;10(13):2824. doi: 10.3390/jcm10132824 (PMC8268164; doi:10.3390/jcm10132824)
Supplement: Supplementary file 1 [file jcm-10-02824-s001.zip › Supplementary data_final version after publish.pdf]

Review

The Antihypertensive Effects and Safety of LCZ696 in Patients with Hypertension: A Systemic Review and Meta-Analysis of Randomized Controlled Trials

Su-Kiat Chua [1,2,3,†], Wei-Ting Lai [2,3,†], Lung-Ching Chen [2,3] and Huei-Fong Hung [2,3,*]

[1] School of Medicine, College of Medicine, Fu Jen Catholic University, New Taipei 24205, Taiwan; M006507@ms.skh.org.tw
[2] Division of Cardiology, Department of Internal Medicine, Shin Kong Wu Ho-Su Memorial Hospital, Taipei 111045, Taiwan; M011645@ms.skh.org.tw (W.-T.L.); M010281@ms.skh.org.tw (L.-C.C.)
[3] Department of Internal Medicine, Shin Kong Wu Ho-Su Memorial Hospital, Taipei 111045, Taiwan
* Correspondence: M000720@ms.skh.org.tw; Tel.: +886-2-2833-2211 (ext. 2084)
† Both authors contribute equally to this work.

Supplementary Data

Table S1. Search terms used in each database.

**PudMed database, MeSH term**
Angiotensin receptor antagonists/ blockers; Angiotensin II receptor antagonists/ blockers; Anti hypertensive agents/ drugs; Hypertension; LCZ 696; neprilysin
**Embase database**
Hypertension, high blood pressure, angiotensin receptor blockers, angiotensin receptor antagonists, angiotensin II receptor antagonists, angiotensin II receptor blockers, LCZ696, ARNI, angiotensin receptor neprilysin inhibitor
**Cochrane database**
Hypertension, high blood pressure, angiotensin receptor blockers, angiotensin receptor antagonists, angiotensin II receptor antagonists, angiotensin II receptor blockers, LCZ696, ARNI, angiotensin receptor neprilysin inhibitor

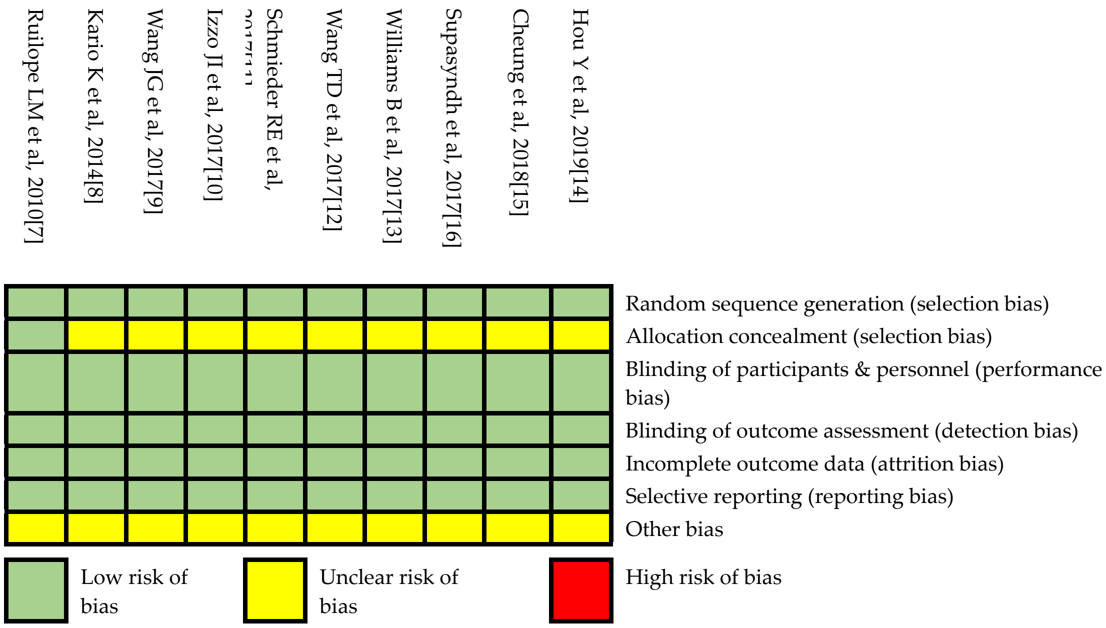

Figure S1. Methodological quality assessment.

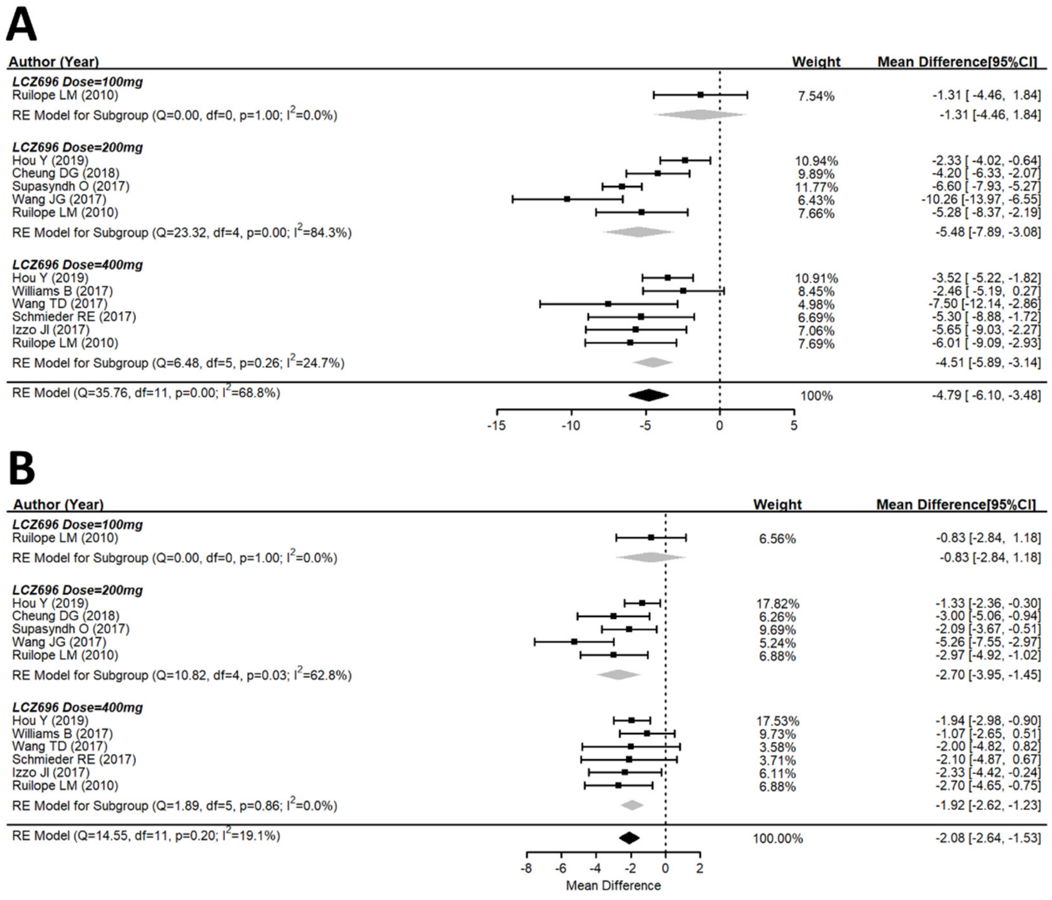

Figure S2. Forest plot of (A) msSBP and (B) msDBP. Comparisons of LCZ696 with a control group. msSBP, mean sitting systolic blood pressure; msDBP, mean sitting diastolic blood pressure.

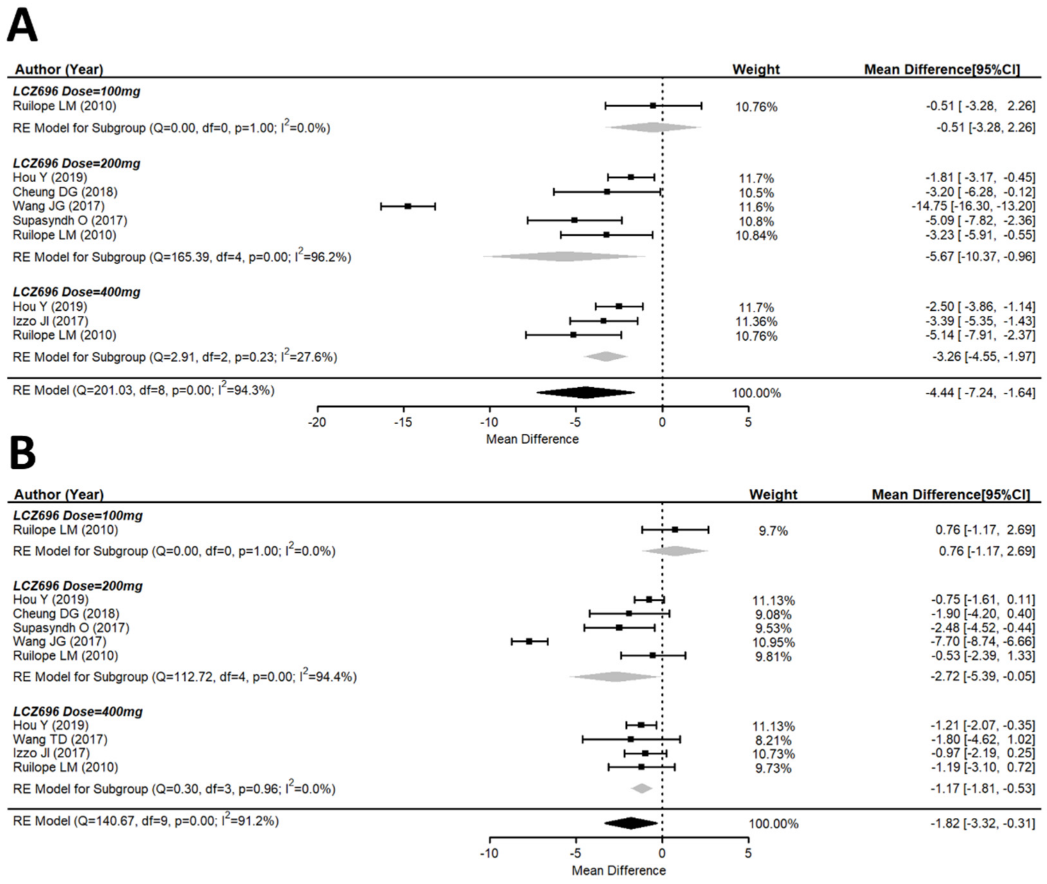

Figure S3. Forest plot of (A) maSBP and (B) maDBP. Comparisons of LCZ696 with a control group. maSBP, mean ambulatory systolic blood pressure; maDBP, mean ambulatory diastolic blood pressure.

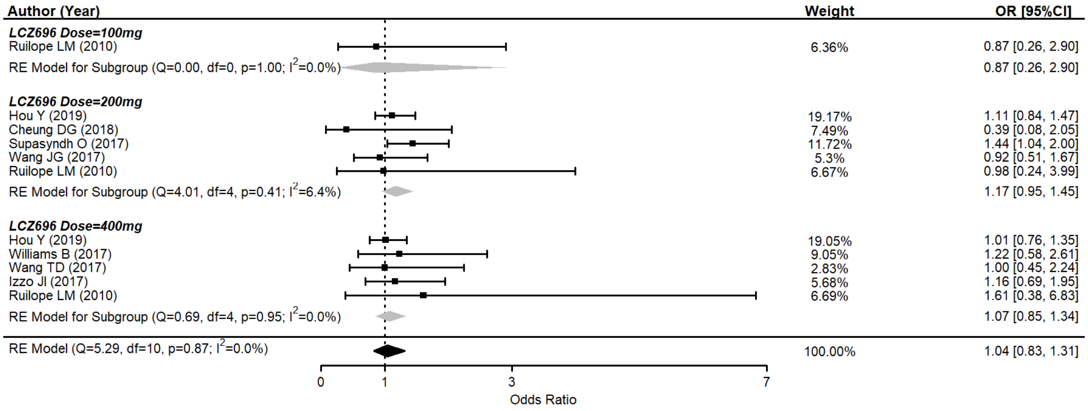

Figure S4. Forest plot of trial-defined adverse events. Comparisons between LCZ696 with a control group.

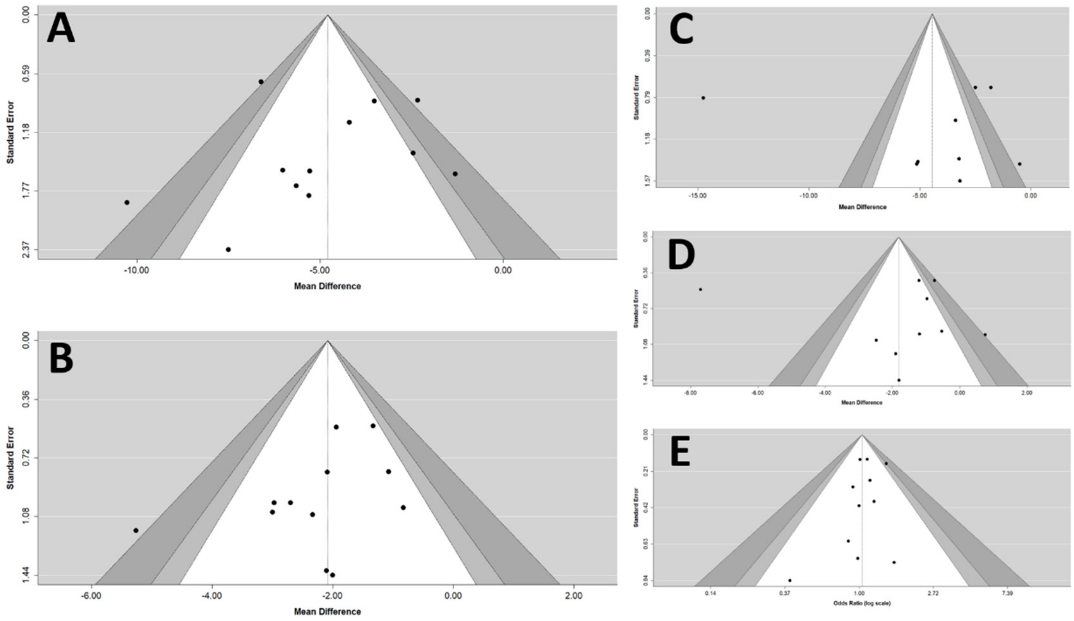

Figure S5. Funnel plots of (A) msSBP, (B) msDBP, (C) maSBP, and (D) maDBP. maSBP, mean ambulatory systolic blood pressure; maDBP, mean ambulatory diastolic blood pressure; msSBP, mean sitting systolic blood pressure; msDBP, mean sitting diastolic blood pressure.
